# A QoS Prediction Approach Based on Truncated Nuclear Norm Low-Rank Tensor Completion

**DOI:** 10.3390/s22166266

**Published:** 2022-08-20

**Authors:** Hong Xia, Qingyi Dong, Jiahao Zheng, Yanping Chen, Cong Gao, Zhongmin Wang

**Affiliations:** 1 School of Computer Science and Technology, Xi’an University of Posts and Telecommunications, Xi’an 710121, China; 2 Shaanxi Key Laboratory of Network Data Analysis and Intelligent Processing, Xi’an 710121, China; 3 Xi’an Key Laboratory of Big Data and Intelligent Computing, Xi’an 710121, China

**Keywords:** QoS prediction, tensor completion, truncated nuclear norm, collaborative filtering

## Abstract

With the rise of mobile edge computing (MEC), mobile services with the same or similar functions are gradually increasing. Usually, Quality of Service (QoS) has become an indicator to measure high-quality services. In the real MEC service invocation environment, due to time and network instability factors, users’ QoS data feedback results are limited. Therefore, effectively predicting the Qos value to provide users with high-quality services has become a key issue. In this paper, we propose a truncated nuclear norm Low-rank Tensor Completion method for the QoS data prediction. This method represents complex multivariate QoS data by constructing tensors. Furthermore, the truncated nuclear norm is introduced in the QoS data tensor completion in order to mine the correlation between QoS data and improve the prediction accuracy. At the same time, the general rate parameter is introduced to control the truncation degree of tensor mode. Finally, the prediction approximate tensor is obtained by the Alternating Direction Multiplier Method iterative optimization algorithm. Numerical experiments are conducted based on the public QoS dataset WS-Dream. The results indicate that our QoS prediction method has better prediction accuracy than other methods under different missing density QoS data.

## 1. Introduction

With the rapid growth of the Internet of Things (IoT) has placed new demands on cloud computing facilities: low latency, high storage, and high bandwidth [1,2]. These demands become more important as more and more services provided by mobile devices are moved to the cloud for storage and applications. However, present cloud computing [3] is not capable of satisfying these new demands. Mobile edge computing (MEC) [4] is a supplement to cloud computing, and edge servers also serve as service carriers to mobile users. The MEC mainly offers mobile services for mobile users who are close to edge computing servers. It solves the shortcomings of insufficient storage capacity, high latency, and low bandwidth for traditional applications to serve users.

With the fast development of MEC, an increasing number of MEC services with similar functionality are appearing at the edge of the network [5]. The main challenge now is to select the most satisfying and suitable service for the user from a very large number of mobile services. This selection of the most appropriate service from a large number of candidate services is called information overload. Therefore, recommending high-quality services for users has become an urgent need. Service recommendations now take into account two main factors: functional and non-functional. The functional contains detailed functional information of a service. The non-functionality generally refers to Quality of Service (QoS) [6]. With the increase of MEC services with similar functions, the non-functional attributes QoS becomes the key foundation for MEC service recommendation and MEC service composition [7]. MEC service QoS contains many attributes such as failure probability, response time, availability, throughput, price, popularity, and more. However, in a real MEC service invocation environment, due to time and network instability, users’ QoS data feedback results are limited, which greatly reduces the user satisfaction and denies the benefits of MEC. So far, service recommendation technology based on QoS prediction has been widely used in Web services, effectively solving the problem of information overload [8]. However, studies involving applications in the field of MEC are few. To combat the issue, QoS prediction for MEC services must be conducted [9].

**Figure 1:**
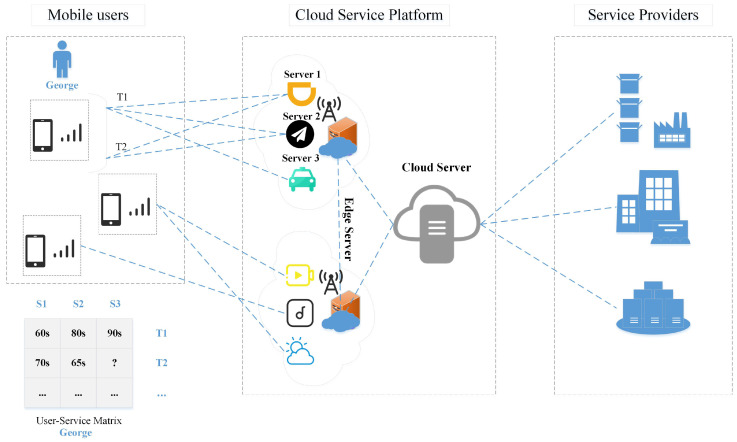
The MEC service invocation scenario.

A real taxi service invocation scenario is shown in Figure 1. The left part is the user, the middle is the cloud server and its services, and the right is the major service providers. The table at the bottom shows the Qos generated by user George invoking the service. The server provides corresponding services according to user requests. As an example for George, the user in Figure 1, suppose that George, a mobile user, wants to invoke a car rental service provided by a service provider uploaded to a cloud server. Thus, the edge server near George provides three candidate services Server 1, Server 2, and Server 3 to George’s mobile device phone. For George, who wants to choose a rental car service, it is difficult to choose between the three rental car services. QoS is an important metric to differentiate the quality of service between functionally equivalent MEC services. In a real invocation scenario, due to a large number of candidate services, in the past, users usually invoked a limited number of MEC services and assigned the corresponding QoS values (car rental services are selected based on QoS an attribute response time). The edge server records that previously, George called services Server1, Server 2, and Server 3 at T1, but called Server1 and Server 2 at T2, resulting in the QoS value of Server 3 being unavailable at T2. Usually, not every user invokes every service provided by the server at every moment. In the case of sparse QoS data, it is difficult to perform relevant operations on MEC mobile services (service recommendation and service composition) [10]. This paper focuses on how to predict missing QoS data values, because QoS record prediction of missing values is a critical step in operations related to mobile services.

In recent years, the problem of predicting missing values in service QoS has received extensive attention and is considered a key factor in service selection. Recommending services to users based on QoS data directly depends on the prediction accuracy of the QoS data. At this stage, many QoS-based service recommendation methods have been developed. Collaborative Filtering (CF) is a fairly mature technology that is widely used in various recommendation scenarios and has achieved good results. In service recommendation applications, CF has been proven to be an effective solution to the problem of information overload in service recommendations. Typical CF methods fall into two categories: neighborhood-based and model-based [11]. The neighborhood-based CF method [12,13] involves simply predicting QoS data based on similar users or services. However, in the real MEC platform invocation service environment, QoS data are often highly sparse, making it challenging to make predictions based on similar users and services. The matrix decomposition method in the model-based CF method [14,15,16] is to obtain two low-dimensional target matrices by learning latent factors. This approach only considers second-order data structures. Thus, considering the time factor that heavily affects QoS data, tensors need to be introduced to model existing QoS records. Although some studies have applied tensor decomposition to QoS prediction [17,18,19], these methods still have shortcomings in that they only consider the global structure of existing QoS records and do not consider the local relationships of QoS, so the accuracy of QoS prediction is not ideal. Therefore, for the low-rank characteristic of QoS data, we propose a model-based CF method to solve the shortcomings of existing methods. The main contributions of this paper are summarized as follows:

1. In order to better express complex multivariate QoS data, we add the dimension of time based on the second-order “User-Service” to form a third-order tensor “User-Service-Time” to represent the QoS data. The QoS data tensor with the addition of time information can well express the complex ternary relationships between data;
2. In order to greatly exploit the QoS data correlation between MEC services, we propose the TLTC (Truncated nuclear norm Low-rank Tensor Completion) method to predict the QoS data. As the constructed QoS third-order tensor has low-rank characteristics, the TCTL method approximates the rank of a tensor by the truncated nuclear norm. Meanwhile, a general truncation rate parameter is introduced to control the degree of truncation of the tensor model in order to better analyze the potential characteristics of the QoS data tensor. Finally, the Alternating Direction Method Multiplies (ADMM) method is used for iterative optimization;
3. In order to prove the effectiveness of the QoS data prediction model based on the TLTC method proposed in this paper, we conducted an experimental evaluation on the public dataset WS-Dream. The metrics use Mean Absolute Error (MAE) and Root Mean Squared Error (RMSE) to evaluate the prediction accuracy. Experimental results show that our QoS data prediction model outperforms other prediction methods.

The organizational framework of this paper is divided into the following sections. Section 2 introduces the related work of QoS data prediction. Section 3 introduces the relevant preparatory knowledge of the tensors involved in this article. Section 4 introduces the QoS data prediction framework and TLTC method proposed in this paper. Section 5 evaluates the proposed method through extensive comparative experiments. Section 6 summarizes the work of the full text.

## 2. Related Work

In this section, related work on QoS data prediction is briefly reviewed. This section also introduces the concept of Low-rank tensor completion and its application in time-series data prediction.

In recent years, QoS data prediction has received a lot of attention, and many QoS data prediction methods have been developed. Accurate prediction of missing QoS values mostly employs Collaborative Filtering methods. A typical neighborhood-based method is the CF method proposed by UPCC [20], which is based on user neighborhood. It measures the correlation between users through a Pearson’s correlation coefficient and predicts missing values using historical QoS data of similar users. This method only considers the relationship between users and does not associate services or time. Moreover, by analyzing the QoS dataset, it is concluded that the number of users is small compared to the number of services, so the overall prediction effect cannot meet the users’ needs. The work in [21] proposes an improved CF algorithm for adjacent user sites. It overcomes the disadvantage of low recall. In order to better capture the correlation between services. The authors of [22] and [23] propose the IPCC and UIPCC methods, respectively. Similar to the UPCC method, the IPCC method uses a parameter to measure the correlation between services. The UIPCC method is a simple combination of the two methods to integrate the prediction results. Furthermore, the QoS value is closely related to the temporal context to introduce time into the QoS prediction. This is also a kind of neighborhood-based method. The authors of [24] proposed a neighborhood-based CF method combined with time series for prediction. This method uses a feedback mechanism to compensate for the deficiencies of the predictive model. The work in [25] proposed a similar user-based CF method to capture temporal features in similarity computation. However, the high sparsity of QoS records in a real MEC environment makes it difficult to select similar users and services at a specific time.

The model-based method is different from the nearest neighbor-based CF method, which requires the use of methods such as machine learning and data mining, and deep mining of potential relationships between data, for example, building a predictive model to predict missing values based on users’ historical preference data for items [26]. Among them, the Probabilistic Matrix Factorization (PMF) [14] method in the model based CF has been successfully applied to the QoS prediction of Web services. The PMF models QoS data as a “User-Service” matrix, where each item in the matrix is a user-generated QoS value for a set of services, which use the Gaussian assumption to decompose the user service quality matrix to predict missing QoS values. The adaptive matrix factorization method is proposed in [27]. This method trains the matrix decomposition model through data transformation and adaptive weighting and predicts the QoS value of user demand services. Although matrix factorization-based QoS prediction methods are generally better than traditional neighborhood-based QoS prediction methods, there are still some shortcomings that do not consider the impact of time factors on QoS data. The non-negative tensor decomposition model is proposed in [18]. A data tensor “user-service-time” model is established using tensor CANDECOMP/PARAFAC (CP) decomposition, and non-negative constraints are added to the decomposition process. The authors of [19] proposed a service prediction method based on tensor decomposition that uses the mean value of the objective function for regularization. The decomposition method based on the tensor decomposition model considers the role of time information. However, in the process of tensor decomposition, the use of tensor decomposition results in a large amount of calculation and data loss. Furthermore, the non-convex relaxation of ranks is intractable, which means it is difficult to recover the CP rank tensor. Therefore, there is still room for improvement in prediction accuracy.

In addition, substantial research has been conducted in data prediction for Low-Rank Tensor Completion (LRTC) methods. Since the “rank” in tensor decomposition is an NP-Hard problem that is difficult to solve, some studies have solved it by introducing the nuclear norm (NN) as the minimized “rank” function. Many variants have been developed on this basis in recent years, but the NN cannot well approximate the “rank” function to achieve the optimal solution [28]. Therefore, the authors of [29] proposed to use the Truncated Nuclear Norm (TNN) to approximate the “rank” for non-convex optimization for tensor completion, and some studies also proved the advantages of using non-convex functions to approximate the “rank” of the tensor [30]. At the same time, the works in [31,32] uses Low-rank tensors to complete the “rank” approximation for data prediction of traffic flow data with temporal and low-rank characteristics and achieved good results. For example, in [33], the TNN approximate tensor “rank” method is used for prediction. Truncated nuclear norm regularization is leveraged to mine structural attributes of tensors and correlations between adjacent elements.

Overall, the fundamental challenge of missing data prediction is to describe correlations and dependencies in the data. Time series data are unique because of their strong similarity and periodicity, and time-series data have a certain temporal correlation and low-rank properties [34]. To better capture QoS correlations and take advantage of the global structure across time, data-missing values are predicted by building the data into tensors. In general, the original second-order “User-Service” matrix is transformed into a third-order “User-Service-Time” tensor by introducing “time” as the time dimension. Inspired by the tensor completion in the prediction of time-series data, we propose a TLTC QoS data prediction method for the low-rank characteristics of QoS data. Low-rank tensor completion methods have demonstrated better predictive accuracy than other methods [35].

## 3. Preliminaries

This section introduces the concept of tensors and some operations on tensors. The evolution of the basic models of LRMC and LRTC is then detailed.

### 3.1. Expression of Tensors

Tensors are higher-order generalizations of arrays, and in recent years, they have emerged as powerful analytical tools for data representation. Now, the use of tensors to express and process high-dimensional data has received extensive attention. It can better express the complex relationships of multivariate data. This section only introduces the background knowledge of tensors used in the paper. Generally, a vector is a first-order tensor, which is represented by bold and lowercase letters x,y. A matrix is a second-order tensor, which is represented by X,Y, such as matrix $X\in R^{I_{1}\times I_{2}}$. Scalars denote *x*, *y* with lowercase letters. The tensors can be represented by bold Euler letters X,Y, then an N-order tensor can be represented as $X\in R^{I_{1}\times I_{2}\times I_{3}\times \cdots \times I_{N}}$.

**Figure 2:**
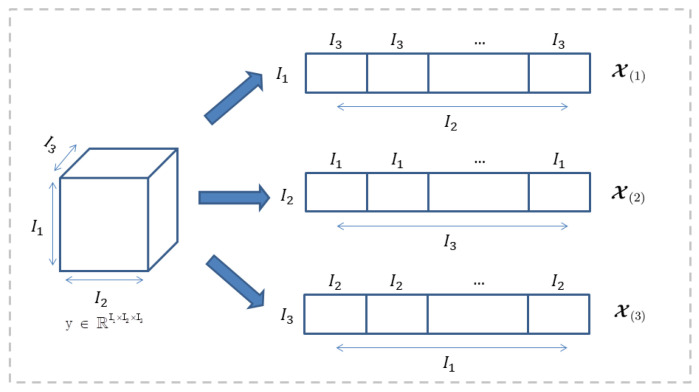
Tensor module unfold.

Tensor unfold: The tensor unfolds along the n-th called tensor matricization. Tensor matricization is the unfolding of tensors into a matrix format with a predefined pattern ordering. Figure 2 shows unfolding of the tensor, matrices $X_{\left(1\right)}\in R^{I_{1}\times I_{2}I_{3}}$,$X_{\left(2\right)}\in R^{I_{2}\times I_{3}I_{1}}$ and $X_{\left(3\right)}\in R^{I_{3}\times I_{1}I_{2}}$ are tensors respectively, 1, 2, 3 modules are unfolded. At the same time, flod(·) is used to convert a matrix into a k-th order high-order tensor $fold_{k}\left(X_{\left(k\right)}\right)=X$.

Frobenius-norm: Assuming a matrix **X** is given, its Frobenius norm is defined as ${∥X∥}_{F}=\sqrt{\sum_{ij}x_{ij}^{2}}$, which is the square root of the sum of all elements. A tensor is an extension of a matrix, given a tensor X, then the Frobenius norm is defined as ${∥X∥}_{F}=\sqrt{\sum_{i_{1},i_{2},\cdots ,i_{n}}x_{i_{1},i_{2},\cdots ,i_{n}}^{2}}$.

Tensor inner product: Given two tensors, when the order of the two tensors is the same, the inner product is a number. Thus follows Equation (1):

(1) $$L=\sum_{i_{1},i_{2},\cdots ,i_{n}}^{I_{1},I_{2},\cdots ,I_{n}}x_{i_{1},i_{2},\cdots ,i_{n}}y_{i_{1},i_{2},\cdots ,i_{n}}$$

### 3.2. Low-Rank Matrix Completion

First, we introduce the concept of Low-rank matrix completion (LRMC). For a partially observable matrix $Y\in R^{I_{1}\times I_{2}}$, the LRMC model is presented in Equation (2):

(2) $$\begin{array}{cc} \; & \min_{X}:rank\left(X\right)\\ \; & s.t.X_{\Omega }=Y_{\Omega } \end{array}$$

where $X_{\Omega }\in R^{I_{1}\times I_{2}}$ is the recovered matrix that we hope to find, and Ω is the index set of observed elements, where rank(X) is the algebraic rank of the matrix **X**. The matrix rank minimization problem in the formula is NP-Hard, as the function is a non-convex function. The most common method is to use the NN to approximate the rank of the matrix, which can be transformed into a convex optimization problem. Equation (2) can be transformed into Equation (3):

(3) $$\begin{array}{cc} \; & \min_{X}:{∥X∥}_{*}\\ \; & s.t.X_{\Omega }=Y_{\Omega } \end{array}$$

where ${∥X∥}_{*}=\sum_{i=1}^{\min(m,n)}\delta_{i}$ is the nuclear norm, $\delta_{i}$ is i-th singular value of the matrix.

### 3.3. Low-Rank Tensor Completion

Tensors are generalized from higher-order matrices. The Low-rank tensor completion (LRTC) is a general tensor completion method. The prior condition is to assume that the elements of this tensor are of low rank in the partial observations. This is the same as the LRMC algorithm. Given a third-order tensor $Y\in R^{U\times S\times T}$, the model of LRTC is:

(4) $$\begin{array}{cc} \; & \min_{X}:rank\left(X\right)\\ \; & s.t.P_{\Omega }\left(X\right)=P_{\Omega }\left(Y\right) \end{array}$$

$X\in R^{U\times S\times T}$ is the tensor to be recovered, and Ω is the index set of observed entries. For tensors, the minimization of tensor rank is an NP-Hard problem that cannot be computed, just like the minimization of matrix rank. To solve this problem, the same solution as matrix completion uses the NN approximation to minimize. Therefore, the above Equation (4) can be transformed into Equation (5):

(5) $$\begin{array}{cc} \; & \min_{X}:{∥X∥}_{*}\\ \; & s.t.P_{\Omega }\left(X\right)=P_{\Omega }\left(Y\right) \end{array}$$

The nuclear norm of the tensor ${∥X∥}_{*}=\sum_{k=1}^{3}\alpha_{k}{∥X_{\left(k\right)}∥}_{*}$. Where $\alpha_{k}>0\left(k=1,2,3\right)$ represents the weight parameter.

## 4. Prediction Framework and Method

### 4.1. Prediction Framework

**Figure 3:**
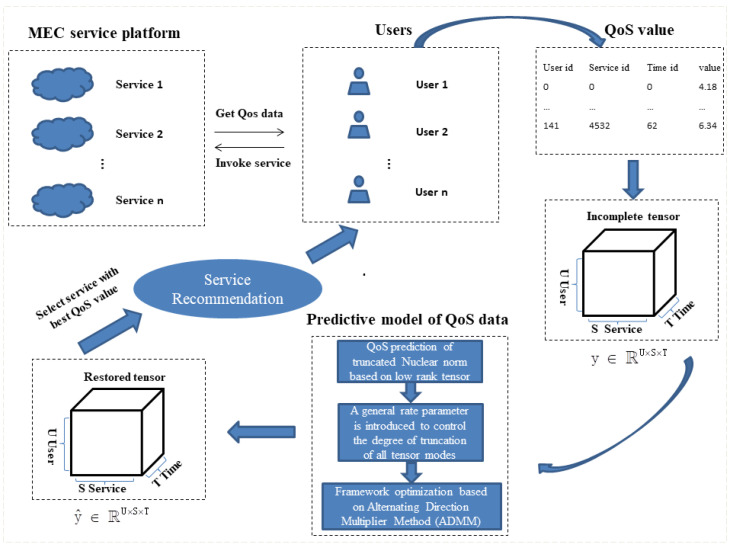
QoS prediction model based on TLTC.

In the real MEC mobile service application environment, the QoS data and the value declared by the provider will be slightly different. Usually, due to time and network constraints, only limited QoS data are available to each user calling the service. It is difficult to provide users with high-quality services and accurate recommendations using these missing QoS data. Therefore, we propose a TLTC-based QoS prediction framework for the low-rank characteristics of QoS data to predict the lost values of QoS data. Figure 3 shows the QoS data prediction framework model proposed in this paper. First, the user obtains sparse QoS data from the server. After data processing, a tensor data model is constructed. After the training and optimization of the TLTC model, the complete recovered QoS data are obtained. Then, the predicted QoS values are sorted, and the service that meets the user’s needs is recommended to the user.

**Figure 4:**
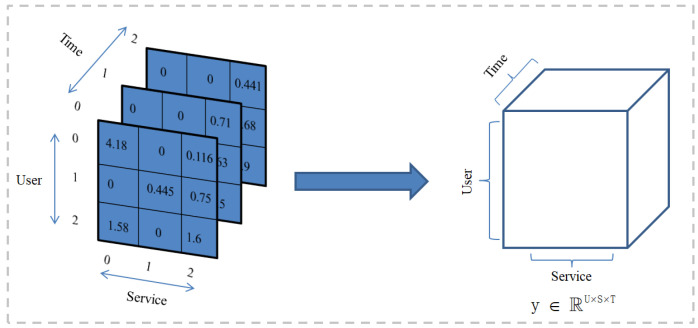
Construction of “User-Service-Time” Tensor Model.

When a user invokes a service, it takes time to accumulate. The QoS values of the services invoked by users are recorded to form a series of QoS datasets. The QoS attributes data set is represented as <UserID,ServicID,TimeID,Value>. As shown in Figure 3, the collected data are sparse. As shown in Figure 4, we construct the QoS data into a third-order QoS data tensor. Specifically, it is assumed that the user whose User ID is 0 invokes the service whose Service ID is 0 when the Time ID is 0 and 2. The user whose User ID is 0 invokes the service whose Service ID is 2 when the Time ID is 0, 1, and 2. Therefore, the QoS values obtained by the same service invoked by the user in multiple time intervals can be stored in the data tensor model as time series. The obtained QoS data are constructed as a third-order tensor $Y\in R^{U\times S\times T}$, and the missing values are filled with 0. The specific construction of the tensor algorithm is as in Algorithm 1. The key task of our paper is to predict the unknown QoS value in the above third-order tensor.

Algorithm 1 presents the construction of the QoS data tensor model. The input is the collected sparse QoS data quadruple <UserID,ServicID,TimeID,Value>. The output is the QoS data tensor “User-Service-Time” $Y\in R^{U\times S\times T}$ with time information added.
**Algorithm 1:** Tensor Construction “User-Service-Time”.  1:Load all QoS value quadruplets (user, service, time, value).  2:Use the set of (user, service, 1, value) construct a (user, service) matrix $N^{\left(1\right)}$ that takes all *I* users as the rows and all *J* services as the columns at the time   interval 1.  3:Set the elements of the matrix $N^{\left(1\right)}$ value with QoS value.  4:Reconstruct all the matrices $N^{\left(1\right)},N^{\left(2\right)},\cdots ,N^{\left(K\right)}$ for *K* time periods.  5:Return $Y\in R^{U\times S\times T}$

### 4.2. Method

The TLTC method is derived from the matrix truncated nuclear norm as a basis. Let us first introduce the definition of the TNN of a matrix. Suppose a matrix $Z\in R^{I_{1}\times I_{2}}$ and non-negative integer $r<min(I_{1},I_{2})$ are given, *r* is the rank of the matrix. The TNN of the matrix Z can be defined as the sum of the smallest singular values of $min(I_{1},I_{2})-r$. In this definition, large singular values 1 to r in the matrix are not taken into account in truncation, as in Equation (6).

(6) $$\begin{array}{c} {∥Z∥}_{r,*}=\sum_{i=r+1}^{min\{I_{1},I_{2}\}}\delta_{i}\left(Z\right) \end{array}$$

where $\delta_{i}\left(Z\right)$ represents the i-th singular value of the matrix. The collation to follow is: $\delta_{1}\geq \delta_{2}\geq \delta_{3}\geq \cdots \geq \delta_{min\{m,n\}}$.

The definition of TNN for matrices cannot be directly applied to high-dimensional tensors. Therefore, we refer to [28,33] for the definition of tensor truncation nuclear norm. As the basis of LRTC for TNN minimization, the truncation of each tensor mode will be automatically assigned if the rate parameter can be set appropriately. For the tensor Z, the TNN definition model of the tensor follows Equation (7):

(7) $$\begin{array}{c} {∥Z∥}_{\gamma ,*}=\sum_{k=1}^{d}\alpha_{k}{∥Z_{\left(k\right)}∥}_{r_{k},*} \end{array}$$

where the truncation for each tensor mode is: $r_{k}=\left\lceil \gamma \cdot min\{n_{k},\prod_{h\neq k}n_{h}\}\right\rceil ,k\in \left\{1,2,3,\cdots ,d\right\}$.

Where *d* represents the mode of tensor unfold. The parameter γ is a general rate parameter that controls the degree of truncation of the tensor *d* modulo, where $r_{k}$ should be satisfied with $1\leq r_{k}\leq min\left\{n_{k},\prod_{h\neq k}n_{h}\right\}$, and $\alpha_{1},\alpha_{2},\alpha_{3},\cdots ,\alpha_{d}$ represents the weight parameters for the tensor Z when it is expanded into a matrix to calculate TNN.

According to the definition of tensor TNN, the model of TLTC for QoS data tensor $Y\in R^{U\times S\times T}$ can be into Equation (8):

(8) $$\begin{array}{cc} \; & \min_{Z}\sum_{k=1}^{3}\alpha_{k}{∥Z_{\left(k\right)}∥}_{r_{k},*}\\ \; & s.t.P_{\Omega }\left(Z\right)=P_{\Omega }\left(Y\right) \end{array}$$

Prevent the dependency of its variables from being guaranteed when the tensor is unfolded. Add an extra set of constraints to this. Introduce auxiliary tensor G. Under certain conditions $Z_{k}=G,k=1,2,3$. For this, the TLTC model is transformed following Equation (9):

(9) $$\begin{array}{cc} \; & \min_{G,Z_{1},Z_{2},Z_{3}}\sum_{k=1}^{3}\alpha_{k}{∥Z_{k(k)}∥}_{r_{k},*}\\ \; & s.t.\left\{\begin{array}{cc} \; & Z_{k}=G,k=1,2,3,\\ \; & P_{\Omega }\left(G\right)=P_{\Omega }\left(Y\right) \end{array}\right. \end{array}$$

For the optimization algorithm, the ADMM algorithm is used for iterative optimization. The ADMM framework optimally solves each variable similarly. Derive the augmented Lagrangian function of formula of Equation (10).

(10) $$\begin{array}{c} L\left(G,{\left\{Z_{k},T_{k}\right\}}_{k=1}^{3}\right)=\sum_{k=1}^{3}\left(\begin{array}{c} \alpha_{k}{∥Z_{k(k)}∥}_{r_{k},*}+\frac{P_{k}}{2}{∥Z_{k(k)}-G_{\left(k\right)}∥}_{F}^{2}+\langle Z_{k}-G_{k},T_{k}\rangle \end{array}\right) \end{array}$$

Therefore, ADMM optimization iteration has converted the tensor prediction problem into the following three solving problems:

(11) $$\begin{array}{cc} \; & Z_{k}^{l+1}=arg\min_{Z}L\left(G,{\left\{Z_{k}^{l+1},T_{k}^{l}\right\}}_{k=1}^{3}\right),\\ \; & G_{k}^{l+1}=arg\min_{G}L\left(G,{\left\{Z_{k}^{l+1},T_{k}^{l}\right\}}_{k=1}^{3}\right),\\ \; & T_{k}^{l+1}=T_{k}^{l}+\rho_{k}\left(Z_{k}^{l+1}-G^{l+1}\right), \end{array}$$

The relationship order of these three variables is derived as: $Z_{1}^{l+1}\to \cdot \cdot \cdot \to Z_{3}^{l+1}\to G^{l+1}\to T_{1}^{l+1}\to \cdot \cdot \cdot \to T_{3}^{l+1}$. Let Equation (11) $\rho_{k}=\rho$, the variable $T_{k}^{l+1}$ is converted to: $T^{l+1}:=T^{l}+\rho \left({\tilde{Z}}^{l+1}-{\tilde{G}}^{l+1}\right)$. Where $T,\tilde{Z},\tilde{G}$ is a fourth-order tensor. Please refer to the [33] if there are too many explanations.

In the following, we provide the derivation formulae for the variables $Z_{k}^{l+1}$ and $G_{k}^{l+1}$.

(12) $$\begin{array}{cc} \; & Z_{k}^{l+1}=arg\min_{Z}\alpha_{k}{∥Z_{\left(k\right)}∥}_{r_{k},*}+\frac{\rho_{k}}{2}{∥Z_{\left(k\right)}-G_{\left(k\right)}^{l}∥}_{F}^{2}+\langle Z_{\left(k\right)},T_{k(k)}^{l}\rangle \\ \; & =arg\min_{Z}\alpha_{k}{∥Z_{\left(k\right)}∥}_{r_{k},*}+\frac{\rho_{k}}{2}{∥Z_{\left(k\right)}-\left(G_{\left(k\right)}^{l}-\frac{1}{\rho_{k}}T_{k(k)}^{l}\right)∥}_{F}^{2}\\ \; & =arg\min_{Z}G\left(Z_{\left(k\right)}\right) \end{array}$$

According to the formula in Equation (12), we can iteratively obtain $Z_{k}^{l+1}$. However, the final $G(Z_{\left(k\right)})$ is a non-convex function, and the optimal solution cannot be achieved by using the usual solution. We refer to [33] to derive Equation (13).

(13) $$\begin{array}{cc} \; & Z_{k}^{l+1}:=fold_{k}\left(Udiag\left(\sigma \left(Z_{\left(k\right)}\right)\right)V^{T}\right) \end{array}$$

The $Udiag\left(\sigma \left(Z_{\left(k\right)}\right)\right)V^{T}$ denotes the singular value decomposition of the tensor unfold matrix, where U and V are a matrix.

(14) $$\begin{array}{cc} G^{l+1} & :=arg\min_{G}\sum_{k=1}^{3}\left(\frac{\rho_{k}}{2}{∥Z_{k(k)}^{l+1}-G_{\left(k\right)}∥}_{F}^{2}-\langle G_{\left(k\right)},T_{k(k)}^{l}\rangle \right)\\ \; & =\frac{1}{\sum_{k=1}^{3}\rho_{k}}\sum_{k=1}^{3}\left(\rho_{k}Z_{k}^{l+1}+T_{k}^{l}\right) \end{array}$$

where $P_{\Omega }\left(G^{l+1}\right)=P_{\Omega }\left(Y\right)$ is a fixed constraint, in order to ensure the transformation of observation information at each iteration.

We described the TLTC model and its ADMM optimization algorithm in detail above, as shown in Algorithm 2.
**Algorithm 2:** TLTC Optimization Imputation Algorithm.  1:Initialize $P_{\Omega }\left(G_{k}^{0}\right)=P_{\Omega }\left(Z\right)$.  2:Set parameter $\rho_{k}$, $\alpha_{k}$, γ, *l* = 0, $T^{0}=0$.  3:While not converged do  4:$\rho =min\{1.05\times \rho ,\rho_{max}\}$  5:For k = 1, 2, 3 do  6:Update $Z_{k}^{l+1}\leftarrow Equation\left(13\right)$  7:EndFor  8:Update $T^{l+1}$  9:Update $G^{l+1}\leftarrow Equation\left(14\right)$  10:l=l+1  11:EndWhile  12:Return $\hat{Y}\in R^{U\times S\times T}$

## 5. Experiment Description

In this section, we present experiments on the QoS data of MEC to verify the prediction accuracy of the TLTC method. The QoS data set uses Throughput attributes and Response Time attributes. We use the evaluation metrics MAE and RMSE. Experiments were compared with seven methods in the case of different data density missing rates. At the same time, we experimentally analyze the influence of integer truncation rate parameters and tensor density on the experimental results. As the experimental operations are randomly removed from the data, this affects the stability of the results. In order to avoid the contingency of the experiment, each experiment was run ten times and the average value was taken as the result.

### 5.1. Database and Baseline Models

We use the well-known large-scale data WS-Dream for experiments. We preprocessed the original dataset and constructed a third-order tensor data structure, “User-Service-Time”. The dataset records the Throughput and Response Time data when 142 users invoke 4532 services in 64 intervals (15 min intervals). Both datasets contain 30,287,611 records. The response time indicates the time duration between the service user sending the request and receiving the corresponding response, whereas throughput is indicated as the average rate of successful message size delivery per second over the communication channel. The detailed statistical information about the QoS attributes data is shown in Table 1. This paper mainly studies two representative attributes of Throughput and Response Time in QoS data. However, it can be used to directly predict any other QoS attribute without modification.

In order to better approximate the real calling environment in this paper, we need to delete the QoS dataset randomly to ensure the sparsity of its QoS data. The detailed description is shown in Table 2. In this paper, the density of the training dataset is set between 10% and 30% in increments of 5%. For fairness of the evaluation, we assign the same initial assumptions to the model, and under the same training and testing datasets, MAE and RSME indicators are used to compare the error between the predicted value and the original value.

In order to verify the effectiveness of the algorithm, we choose the most classic seven QoS prediction algorithms to compare the prediction accuracy on the same dataset. The following baseline models are compared UPCC [20], IPCC [22], UIPCC [23], PMF [24], NTF [18], WSPred [19], CLUS [36].

### 5.2. Evaluation Metrics

In order to accurately evaluate the prediction criteria of Qos, the MAE and RMSE are used as the criteria to compare with other baselines. The MAE formula and RMSE formula are Equation (15) [37]:

(15) $$\begin{array}{cc} \; & MAE=\frac{1}{N}\sum_{i,j,k}\left|y_{ijk}-{\hat{y}}_{ijk}\right|,\\ \; & RMSE=\sqrt{\frac{1}{N}\sum_{i,j,k}\left(y_{ijk}-{\hat{y}}_{ijk}\right)}. \end{array}$$

where $y_{ijk}$ represents the real Qos value of the test data set, true QoS value of user *i* calling service *j* at interval *k*. ${\hat{y}}_{ijk}$ represents the predicted Qos value of the training data set after model training. The smaller the MAE and RMSE values, the better the prediction effect.

### 5.3. Parameter Settings

The weight parameter $\alpha_{k}$, the truncation rate parameter γ and the learning rate ρ in ADMM, these three parameters will directly affect the prediction accuracy of the model. The setting of the truncation rate parameter γ can be obtained by cross-validation. The low-rank tensor is feasible for processing time-series data with the same parameter selection as in [31], using cross-validation for selection. We also performed a local analysis while performing cross-validation to select parameters. If cross-validation is used, selecting the appropriate weight parameter $\alpha_{k}$ requires extensive computation. We refer to the parameter setting of the HaLRTC model in [28] and set the parameter to $\alpha_{1}=\alpha_{2}=\alpha_{3}=1/3$. The ρ parameter is learned in ADMM, which determines the convergence of the entire model. We set $\rho =1\times 10^{-5}$ and $\rho =1\times 10^{-4}$ in the two QoS attribute datasets of Throughput and Response Time, respectively. The maximum number of iterations is set to 20 and 50 to achieve convergence.

### 5.4. Analysis of Results

In the comparison experiments, we set the initial conditions of all algorithmic models to be the same and use the same evaluation criteria and datasets. The Neighborhood-based CF methods such as UIPCC, UPCC and IPCC set their neighborhood users to 10 and the number of neighborhood services to 50. The latent factor matrix dimension is set to 20 in tensor factorization or matrix factorization. Some methods are unable to construct tensors for testing. We use the same special treatment as in [18] to compress the tensor into a “user-service” matrix. We calculated MAE and RMSE for these methods and compared them with our TLTC method.

**Figure 5:**
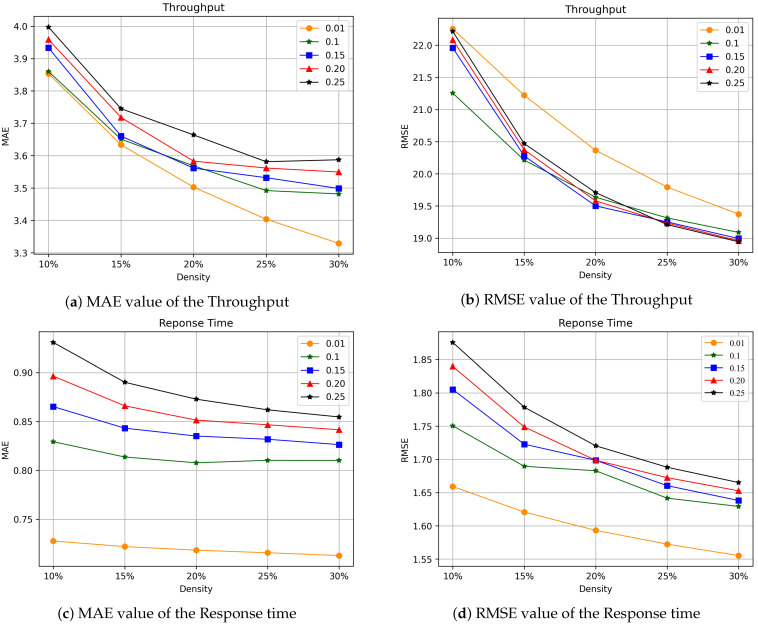
The effect of the truncation rate parameter.

The setting of the truncation rate parameter is selected by cross-validation. However, the prior knowledge of QoS data is relatively small. In order to study the effect of the truncation rate parameter on the performance of the algorithm, the global optimum is guaranteed. This paper refers to the parameter settings of time-series data processing in [26,31]. As shown in Figure 5, we will study the effect of the truncation rate parameter on the prediction accuracy. The tensor density is adjusted from 10% to 30% with a step of 5%. We observe the MAE and RMSE values of Throughput and Response Time for QoS data at different tensor densities.

Figure 5a,b presents the experimental results of the MAE and RMSE of the Throughput. Figure 5c,d presents the experimental results of MAE and RMSE of Response time. First, for the QoS dataset of Throughput attributes, the truncation rate parameters 0.01, 0.1, 0.15, 0.2, 0.25 were used for testing. The number of iterations was chosen to be 20. The experiment shows that when the integer truncation value of the tensor is 0.01, MAE decreases more stably than other values when the density of the tensor is between 10% and 30%. It shows that the difference value is handled well. Moreover, the obtained MAE value is relatively low, indicating that the prediction effect is better when the truncation rate parameter value is 0.01. For the value of RMSE, when the truncation rate parameter is 0.01, although the value is not ideal, the convergence rate is faster than other values. In overall evaluation, the prediction effect is the best when the value of the truncation rate parameter is 0.01. Although the MAE value of the Throughput is relatively low when the value of the truncation rate parameter is 0.1, its RMSE value is relatively high. We should expect MAE values to be much smaller than RMSE values.

For the Qos dataset with Response time attributes, as shown in Figure 5c,d, the parameters 0.01, 0.1, 0.15, 0.2, 0.25 were used for testing, and the number of iterations was 50 converged. It can be observed from the experimental results that, as for the Throughput QoS dataset, when the truncation rate parameter is set to 0.01, the values of MAE and RMSE are the lowest. Therefore, the prediction effect is the most ideal.

Experimental results show that under different tensor densities, the prediction accuracy of our TLTC method has smaller RMSE and MAE values when the truncation rate parameter is 0.01. According to prior experience, the truncation rate parameter is locally verified to achieve the desired prediction accuracy and save a lot of computational costs. With the increase of tensor density, when the truncation rate parameter is 0.01, the prediction accuracy of the TLTC method gradually increases. This means that providing more QoS data can lead to higher prediction accuracy.

**Figure 6:**
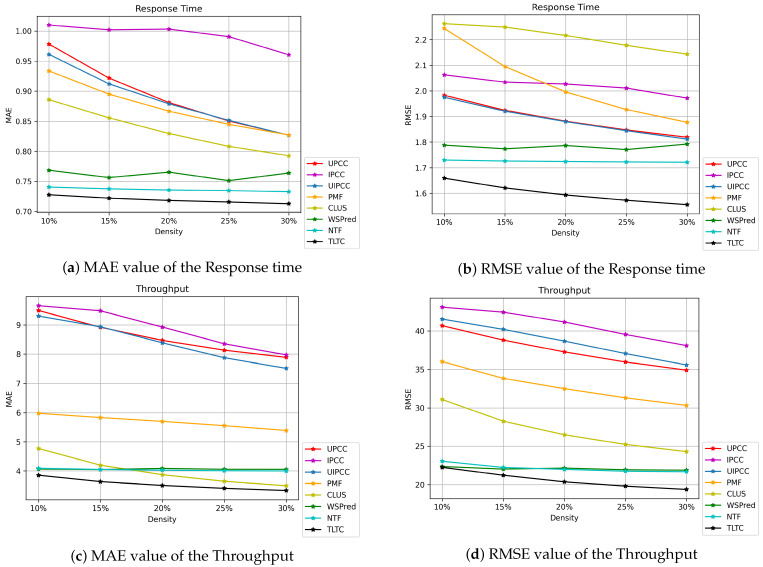
Comparison of QoS prediction accuracy based on MAE and RMSE.

Experimental results show the effectiveness of our TLTC method. We select seven classical prediction methods to compare with the QoS data prediction methods of our TLTC model. In the same training and testing sets, the evaluation metrics MAE and RMSE of the prediction accuracy of these methods are obtained in this paper through extensive experiments as shown in Figure 6. Figure 6a,b shows the results of MAE and RMSE values for the response time. Figure 6c,d presents the MAE and RMSE results of Throughput. As can be seen from Figure 6, the neighborhood-based CF methods UPCC, IPCC, UIPCC and matrix factorization PMF methods have lower prediction accuracy than the WSPred, NTF, and TLTC methods using tensor models. Other methods only utilize the second-order static relationship of the “User-Service” model and do not consider the time information in the “User-Service-Time” model. For example, the TLTC framework proposed in this paper significantly improves the prediction accuracy of QoS data, and obtains smaller MAE and RMSE values for both Response Time and Throughput under different QoS data missing densities. For methods that also use the tensor model WSPred and NTF, they also add time information. However, when using tensor decomposition for predicting QoS data, it does not pay much attention to the data loss due to decomposition during prediction, and the prediction accuracy is much worse than expected. We adopt the method of low-rank tensor completion, using the automatic unfold tensor mode with TNN, it can better focus on strong temporal correlation between QoS data. Therefore, the prediction accuracy of our proposed TLTC method is higher than that of other methods. In addition, because the range of Throughput is 0–1000 kbps, and the range of response time is only 0–20 s, the MAE and RMSE for Throughput are much larger than the MAE and RMSE values for Response Time.

The experiments show that the prediction accuracy is positively correlated with the tensor density of QoS data. It can be seen from Figure 6 that the density of the training tensor is changed from 10% to 30%, and the step size increases by 5%, the prediction accuracy of method also gradually changes. The prediction accuracy of the TLTC method increases with the increase of training density. This is because the greater the density of QoS data which provides more useful information, the better the prediction effect of the method.

## 6. Conclusions

In this paper, to better express the global structure of QoS data in the real MEC environment, we add time information to the current QoS data to construct a “User-Service-Time” third-order tensor. To this end, for the low-rank properties of the QoS data tensor, we propose a TLTC method to predict the missing values of QoS data. The TLTC method avoids the disadvantages of data loss and ignoring local data caused by traditional tensor decomposition. The method can better capture the temporal correlation between different users and different services by regularizing the TNN. Considering the hidden correlation of QoS data, a general rate parameter is introduced to control the truncation degree of all tensor patterns. Finally, the low-rank estimate of the target tensor is obtained to predict the missing values of the QoS data using ADMM iterative optimization of the approximation tensor. In this paper, experimental studies on the public large-scale QoS dataset WS-Dream indicate that our method outperforms other methods with higher prediction accuracy.

## Figures and Tables

**Table 1:** The number of services.

Statistics	Throughput	Response Time
Scale of QoS values	0–1000 kbps	0–20 s
Mean of QoS values	9.609 kbps	3.165 s
Standrad Deviation	50.11 s	6.12 s
Num. of Users	142	142
Num. of Services	4532	4532
Num. of Time Intervals	64	64
Interval of Time Slots	15 min	15 min
Num. of Records	30,287,611	30,287,611

**Table 2:** QoS data preprocessing.

No.	Train:Test	Training Data	Testing Data
1	10%:90%	3,028,761	27,258,850
2	15%:85%	4,543,142	25,744,469
3	20%:80%	6,057,522	24,230,089
4	25%:75%	7,571,903	22,715,708
5	30%:70%	9,086,283	21,201,328

## Data Availability

Data sharing not applicable.
